# Supplementary material for: The burden of SARS-CoV-2 among healthcare workers across 16 hospitals of Kashmir, India—A seroepidemiological study
Source: PLoS One. 2021 Nov 19;16(11):e0259893. doi: 10.1371/journal.pone.0259893 (PMC8604293; doi:10.1371/journal.pone.0259893)
Supplement: S1 Table — (DOCX) [file pone.0259893.s001.docx]

S1 Table: Hospital wise participation of HCWs across 16 hospitals

| Hospital | No. of HCWs | No. of HCWs participated |
| --- | --- | --- |
| District hospital Ganderbal | 195 | 104 |
| District hospital Bandipora | 108 | 85 |
| District hospital Badgam | 110 | 78 |
| District hospital Pulwama | 307 | 100 |
| District hospital Shopiyan | 73 | 64 |
| District hospital Baramulla | 174 | 159 |
| District hospital Kupwara | 200 | 187 |
| District hospital Kulgam | 214 | 77 |
| District hospital Anantnag | 206 | 153 |
| JLNM hospital Srinagar | 132 | 120 |
| SMHS & SS hospital Srinagar | 2200 | 244 |
| Chest disease hospital Srinagar | 210 | 43 |
| Maternity hospital Srinagar | 255 | 142 |
| Bone and Joint hospital Srinagar | 215 | 184 |
| Pediatric hospital Srinagar | 217 | 180 |
| IMHANS hospital Srinagar | 95 | 83 |
| Total | 4911 | 2003 (40.8%) |
