# Supplementary material for: The burden of SARS-CoV-2 among healthcare workers across 16 hospitals of Kashmir, India—A seroepidemiological study
Source: PLoS One. 2021 Nov 19;16(11):e0259893. doi: 10.1371/journal.pone.0259893 (PMC8604293; doi:10.1371/journal.pone.0259893)
Supplement: Attachment — Submitted filename: response to reviewer.docx [file pone.0259893.s003.docx]

Dear Editor

Ref: PONE-D-21-21086

Thank you for your valuable comments and suggestions. Kindly find below the pointwise response to the reviewer's comments and queries.

Regards

Mariya Amin Quireishi

Corresponding author

Manuscript ID-PONE-D-21-21086

Reviewer #1: This is an important study on a relevant and timely topic. The data would be useful, although we are now past the second wave in formulating public health policy. I would suggest adding a note on the implications of vaccination status in future studies or longitudinal studies on seroprevalence.

Response: Thank you for your valuable suggestion. It has now been added in the discussion section of the manuscript.

Reviewer #2: This study is a cross-sectional estimate of COVID seroprevalence based on the nucleocapsid IgG antibody test. They invited all the level 2 and 3 hospitals in Kashmir. 2 of them refused the invitation. From those who agreed to participate, they invited willing hospital staff to enroll. This serology survey was conducted from January 14 to 17, at a time when the first wave had ceased, and Vaccines were yet to be started.

What is the total number of HCW in each of the hospitals? What % of the staff population accepted to take part in the study?

Response: Breakup of HCWs in each hospital is tabulated. Overall 40.8% HCWs took part in this study. We have added this information in the manuscript as a supplementary file

| Hospital | No. of HCWs | No. of HCWs participated |
| --- | --- | --- |
| District hospital Ganderbal | 195 | 104 |
| District hospital Bandipora | 108 | 85 |
| District hospital Badgam | 110 | 78 |
| District hospital Pulwama | 307 | 100 |
| District hospital Shopiyan | 73 | 64 |
| District hospital Baramulla | 174 | 159 |
| District hospital Kupwara | 200 | 187 |
| District hospital Kulgam | 214 | 77 |
| District hospital Anantnag | 206 | 153 |
| JLNM hospital Srinagar | 132 | 120 |
| SMHS & SS hospital Srinagar | 2200 | 244 |
| Chest disease hospital Srinagar | 210 | 43 |
| Maternity hospital Srinagar | 255 | 142 |
| Bone and Joint hospital Srinagar | 215 | 184 |
| Pediatric hospital Srinagar | 217 | 180 |
| IMHANS hospital Srinagar | 95 | 83 |
| Total | 4911 | 2003 (40.8%) |

Why randomisation was not done?

Response: This was a cross-sectional study, so randomization does not apply here. I believe the reviewer is asking about random sampling here. We did not perform a random sampling strategy in this study due to time constraints and practical difficulty.

why the sample size was not calculated?
**Response:**  Our results are based on the voluntary participation of HCWs. Our study included 2003 HCWs and the seroprevalence estimates of 26.8 % with a confidence interval of (24.8- 28.8) show that the estimates have pretty good precision.
- What is the mean age of HCWs overall - that refused consent?

Response: Thank you for your query. The information of those who did not participate in the study was not collected. We have included it in the limitation section of the manuscript.

What was the PCR positivity prevalence in those who did not take part?

Response: Thank you for your query. We did not collect the information of those who did not participate in the study.

Comorbidities have not been captured!

Response: Information about comorbidities was not collected

- Was appropriate PPE available for all staff at all times? Did the Housekeeping staff get hazmat? A note on the level of preparedness for COVID19 among HCW in Kashmir should be added to help the reader better understand the background.

Response: Thank you. Owing to resource constraints, the PPE was not available for all staff at all times. Hazmut was not provided to the housekeeping staff. A note on the level of preparedness has now been added to the methods section.

Our administrative staff at increased risk of exposure to Clinical areas? Did all the include HCWs work in COVID wards or Emergency services?

Response: Since the study was done 10 months after the appearance of the first case of SARS-CoV-2 infection in Kashmir, the level of exposure among all categories of healthcare workers at this time was not much different. All the hospitals had restarted their routine outpatient and inpatient activities. The same is reflected in our study as well. We did not find any difference in the seroprevalence among different occupational categories of HCWs.
