# Supplementary material for: The burden of SARS-CoV-2 among healthcare workers across 16 hospitals of Kashmir, India—A seroepidemiological study
Source: PLoS One. 2021 Nov 19;16(11):e0259893. doi: 10.1371/journal.pone.0259893 (PMC8604293; doi:10.1371/journal.pone.0259893)
Supplement: Attachment — Submitted filename: response to reviewer.docx [file pone.0259893.s004.docx]

Reviewer #2: This study is a cross-sectional estimate of COVID seroprevalence based on the nucleocapsid IgG antibody test. They invited all the level 2 and 3 hospitals in Kashmir. 2 of them refused the invitation. From those who agreed to participate, they
invited willing hospital staff to enroll. This serology survey was conducted from January 14 to 17, at a time when the first wave had ceased, and Vaccines were yet to be started.

Kindly add the reasons provided by the authors for not calculating sample size and convenience sampling in the methodology section.

Response: Thank you for your suggestion. We have now included it in the methodology section of the manuscript.
